# Steps Toward the Band Gap Identification in Polystyrene Based Solid Polymer Nanocomposites Integrated with Tin Titanate Nanoparticles

**DOI:** 10.3390/polym12102320

**Published:** 2020-10-10

**Authors:** Ahang M. Hussein, Elham M. A. Dannoun, Shujahadeen B. Aziz, Mohamad A. Brza, Rebar T. Abdulwahid, Sarkawt A. Hussen, Sarkawt Rostam, Dalia M. T. Mustafa, Dana S. Muhammad

**Affiliations:** 1 Hameed Majid Advanced Polymeric Materials Research Lab., Department of Physics, College of Science, University of Sulaimani, Sulaimani 46001, Iraq; ahang.hussein@univsul.edu.iq (A.M.H.); sarkawt.hussen@univsul.edu.iq (S.A.H.); 2 Associate Director of General Science Department, Woman Campus, Prince Sultan University, P.O. Box 66833, Riyadh 11586, Saudi Arabia; elhamdannoun1977@gmail.com; 3 Department of Civil engineering, College of Engineering, Komar University of Science and Technology, Sulaimani 46001, Iraq; 4 Department of Manufacturing and Materials Engineering, Faculty of Engineering, International Islamic University of Malaysia, Kuala Lumpur 50728, Malaysia; mohamad.brza@gmail.com; 5 Department of Physics, College of Education, University of Sulaimani, Sulaimani 46001, Iraq; rebar.abdulwahid@univsul.edu.iq (R.T.A.); dana.muhammad@univsul.edu.iq (D.S.M.); 6 Department of Mechanical Engineering/Production, College of Engineering, Sulaimani Polytechnic University, Sulaimani 46001, Iraq; sarkawt.rostam@spu.edu.iq (S.R.); dalia.89@hotmail.co.uk (D.M.T.M.)

**Keywords:** polystyrene, SnTiO_3_ nanoparticle, XRD investigation, UV-Vis investigation, band gap examination, optical properties

## Abstract

In the current study, the film fabrication of polystyrene (PS) based polymer nanocomposites (NCs) with tuned refractive index and absorption edge was carried out using the solution cast method. X-ray diffraction (XRD) and ultraviolet-visible (UV-Vis) light characterization techniques were performed. The structural and optical properties of the prepared films were specified. The hump of PS decreased significantly when SnTiO_3_ nanoparticles (NPs) were introduced. Sharp and high intense peaks of SnTiO_3_ NPs at a high filler ratio were observed. The crystalline size was determined for SnTiO_3_ NPs from the sharp crystalline peaks using Debye-Scherrer’s equation and was found to be 25.179 nm, which is close enough to that described by the supplier. Several optical parameters, such as absorption coefficient (α), refractive index (*n*), and optical dielectric properties, were investigated. The absorption spectra were tuned with increasing SnTiO_3_NPs. Upon the addition of the NPs to the PS host polymer, the absorption edge undergoes shifting to lesser photon energy sides. The optical dielectric constant (ε′) was correlated to the refractive index. The study of the optical band gap was conducted in detail using both Tauc’s model and the optical dielectric loss (ε″) parameter. The results showed that the ε″ parameter is noteworthy to be measured in the optical band gap study of materials.

## 1. Introduction

The study of optical and electrical properties of synthetic and natural polymer-based electrolytes are increasingly in progress because of their complete applications in batteries, capacitors, and fuel cells [1,2,3,4,5,6,7]. Nanotechnology, as a widespread knowledge area, contributes to all fields of science. Recently, nanosize particle incorporation to polymers is identified to be essential for application in optoelectronic and photonic devices. Polymers are examined to be appropriate candidate hosts for inorganic nanoparticles (NPs) [8]. Over the past two decades, the synthesis and examination of inorganic and organic combine materials have escalated intensively and extensively. The concept of combining organic and inorganic materials appears to be proven. However, the complexity of the designs of hybrids has shown relatively low compatibility of the components [9]. The good mechanical, electrical, and optical characteristics of polymer composites are decisive factors for upcoming material uses, for example, flexible photonics or electronics [10]. Light interaction with new materials is essential in optical technologies, from solar cells to light-emitting devices.

Recently, polymers with various optical characteristics have drawn considerable attention of research groups, owing to their general uses in optical devices, sensors, and light-emitting diodes (LEDs). Tuning the materials’ optical characteristics is conducted from changing the identity of dopant materials and optimizing the dopant concentrations [11]. Earlier studies have emphasized that the addition of metals, semiconductor particles, and metal complexes can manipulate the optical and electrical characteristics of polymers [12,13,14,15,16]. Polymer materials in the composite’s form can apply to radio frequency interference and electromagnetic interference shielding and electrostatic dissipation charges. These composites can be employed as electrically conductive adhesives and circuit elements in microelectronics due to possessing anticorrosive behavior [17]. The optical characterizations of polymeric films, such as absorbance, transmittance, and reflectance, are essential to be determined. The optical parameters, for instance, energy gaps, dielectric loss (ε″), dielectric constants (ε′), and extinction coefficient (*K*), are employed in deriving other imperative properties. Here, the optical absorption shifts, i.e., generally the absorption edge shapes and changes, are two promising approaches in understanding the optically induced transitions process in crystalline and noncrystalline materials. To accomplish optoelectronic and photonic devices with higher efficiency, a significant improvement was achieved in understanding the crucial physical and chemical properties of polymers, even though creating a relationship between these two characteristics is not attained yet. Characterizations of polymers’ optical properties, such as optical absorption, infrared dichroism, luminescence spectra, and Raman polarization, are among the vital methods to study electronic characteristics [18,19,20]. Lately, numerous laboratories are supported by many companies, and their works are devoted to creating new materials to be utilized in optoelectronic and photonic devices, for example, organic photovoltaic, LEDS, and polymer lasers [21]. In earlier studies, the optical characteristics of polymers incorporated with Pb(ZrTi)O_3_ and PbTiO_3_ NPs have been studied. However, due to the harmful nature of Pb and its negative effect on the environment, it is highly recommended to substitute Pb with ecofriendly elements [22]. SnTiO_3_ is one of the promising elements that is Pb-free, which was newly investigated [23,24], and it is theoretically shown to have a high dielectric constant (ε_r_) as calculated through the first-principle study [23,24]. Hence, Sn-based materials are known to be a good substitute for Pb-based elements in the upcoming application devices, for instance, piezoelectric power harvesters, transducers, optical waveguides, and nonvolatile memories [24,25,26]. The substitution of the PbTiO_3_ A-site with Sn leads to probable material, which is more environmentally friendly.

In this study, the optical parameters such as refractive index (*n*), *K*, absorption coefficient (α), ε′, and ε″ are determined [11]. In previous studies, it has been confirmed that the electron transition properties from the valence band (VB) to the conduction band (CB) can be examined well from tackling the optical properties of the polymer composites [27,28]. The main properties of the polystyrene (PS) polymer, such as facile processability, relatively inexpensive, and mechanical strength, make it to be a strong candidate for the creation of composite films [29]. The objective of this work is aimed at dealing with the effect of SnTiO_3_ NPs on the optical properties of PS-based polymer composite. The present work shows that the types of electron transitions can be precisely identified with the help of the ε″ parameter. Based on theoretical background and experimental approaches, the parameter of ε″ is efficient for specifying the kinds of electron transitions from Tauc’s model. Thus, the current results of the band gap (BG) analysis will answer several questions that have not still been realized in previous efforts.

## 2. Experimental Details

### 2.1. Hybrid Fabrication

**Scheme 1:**
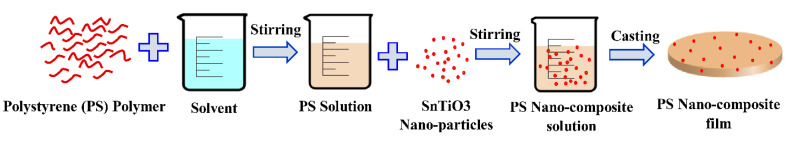
Hybrid fabrication procedure using casting technique.

Sigma-Aldrich (St. Louis, MO, USA) supplied the PS powder material used for our study. SnTiO_3_ nanoparticles (purity 99.9%; Nanografi, Turkey) with a diameter of 30 nm were used with the PS solution to prepare polymer nanocomposites (NCs). Solid polymer nanocomposite PS samples were synthesized by the eminent method of solution cast. The solution of the PS polymer was arranged by incorporating the toluene solvent to the PS powder, stirred by a magnetic stirrer for around 1h. To prepare PS-based nanocomposite films, various amounts of SnTiO_3_NPs were added to the PS polymer solution separately. Schematically, the fabrication methodology is presented in Scheme 1. The PS polymer nanocomposite films were then coded as PSSNT0, PSSNT1, and PSSTN2, corresponding to the PS incorporated with 0, 4, and 8 wt % of SnTiO_3_, respectively. The solutions were cast to different Petri dishes and saved to dry at room temperature for films to form.

### 2.2. X-Ray Diffraction

A D5000 X-ray diffractometer (Malvern Panalytical Ltd., Malvern, UK) operated at 40 kV voltage and 45 mA current correspondingly was used for recording X-ray diffraction (XRD) at room temperature. A monochromatic X-ray radiation beam (λ = 1.5406 Å) and glancing angles (2θ) between 10°and 80° with a 0.05° step size were scanned over the samples.

### 2.3. UV-Vis Measurements

A Jasco V-570 UV-Vis-NIR spectrophotometer (Jasco SLM-468, Tokyo, Japan) in the absorbance mode was used in recording the absorption spectra of the ultraviolet-visible (UV-Vis) light of the nanocomposite films. The thicknesses of films are in the range of 121–123 μm.

## 3. Results and Discussion

### 3.1. XRD Examination

**Figure 1:**
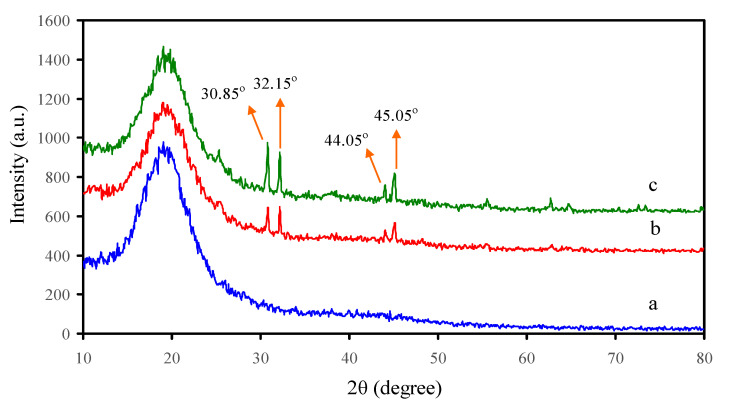
X-ray diffraction pattern for a—pure PS (PSSNT0), b—PSSNT1, and c—PSSNT2 nanocomposite films.

**Figure 2:**
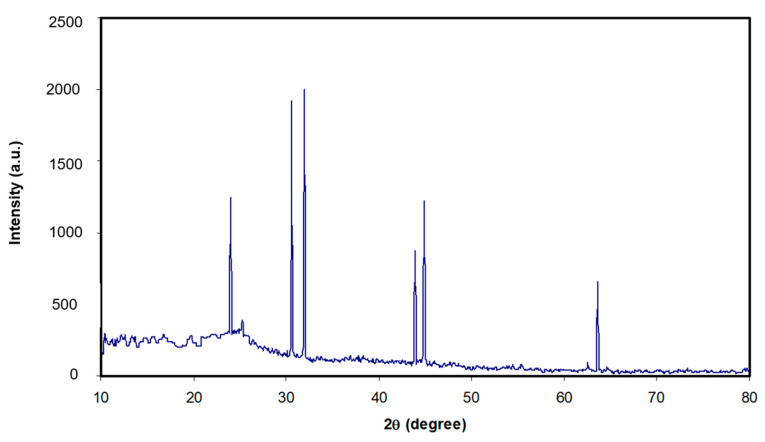
X-ray diffraction pattern for SnTiO3 nanoparticles (NPs).

For the structural analysis, the XRD patterns of the polymer films were recorded. Figure 1 displays the XRD pattern spectra of pure PS and PS NCs at room temperature. It is well known that each polymer has characteristic peaks. To distinguish between crystalline, semicrystalline, and amorphous polymers, the nature of the peak that appears in the XRD patterns decides the degree of crystallinity, where the sharp and broad peaks indicate high crystallinity and amorphous polymers, respectively. For example, poly(ethylene oxide) (PEO) has two sharp peaks at 22° and 18°, in addition to several small intense peaks at higher 2θ degrees, and thus it is identified as a semicrystalline polymer [2,30]. Figure 1 exhibits the XRD pattern of pure PS at room temperature where the amorphous nature of the polymer is indicated from the largest specific diffraction peak at 2θ between 15° and 22° [29,31]. Crystal materials are periodically arranged in 3D space; amorphous materials do not have this periodicity, and atoms are distributed randomly in 3D space. The X-rays would be scattered in specific directions when there is a periodic arrangement of atoms, and consequently, high-intensity peaks can be observed. In the amorphous phase, X-rays would be scattered in various directions, leading to a broad peak distributed in a wide 2θ range instead of high intensity narrower peaks [32]. Thus, pure PS polymer is almost amorphous material due to the absence of sharp crystalline peaks in its XRD pattern. The XRD patterns were collected and analyzed to compare the pure and NC PS films (see Figure 1). It is clear that the crystal structure of PS is moderately altered by SnTiO_3_ NPs’ addition. One study documented that pure PS does not produce sharp peaks, but instead an amorphous halo is produced [33]. Clearly, the main peak of PS is more broadened in NC films. The sharp peaks that appeared in the NC samples are ascribed to the incorporated SnTiO_3_NPs. Bragg peaks in XRD patterns originate in the diffraction of incident X-rays from lattice planes of a crystalline material. The XRD pattern of the SnTiO_3_ NPs exhibited in Figure 2 established that the new peaks which appeared in NC films with weak intensity are ascribed to the incorporated NPs. It is interesting to note that the particle sizes can be estimated using Debye-Scherrer’s formula. The additional diffraction peaks at scattering angles 2θ = 28.25°, 30.65°, 32.05°, 43.95°, and 44.95° of the NC samples are observed, and the intensity of these peaks increased with raising the SnTiO_3_ concentrations. The crystalline sizes were determined using Debye-Scherrer’s equation [34,35]

(1) D = Kλ/β cosθ

where *D* refers to the particle size, λ refers to the X-ray radiation wavelength (λ = 0.154 nm), *k* refers to a constant and typically accepted as 0.9, *β* refers to the full width at half maximum (FWHM) by radian, and *θ* refers to the Bragg angle of the peak. From the outcomes of XRD, the SnTiO_3_ NPs average sizes are assessed to be approximately 25.179 nm as shown in Table 1, and the full width at half maximum values for pure and doped NC samples are presented in Table 2. It can be seen from Figure 1 that the broad peak of the PS host polymer becomes more broadened with increasing SnTiO_3_ concentration. This is evidence for the increase of the amorphous phase in NC films in comparison with the pure PS film. From Equation (1), it is obvious that a larger *β* (FWHM) results in a lower degree of crystallinity. Previous studies established that additions of fillers into the polymer hosts are crucial in influencing the structural, electrical, or mechanical properties of various polymers [36,37,38]. Figure 2 depicts the XRD pattern for pure SnTiO_3_ NPs. Noticeably, the sharp peaks with high intensity can be seen. The decrease of these peaks in Figure 1 reveals some kind of interaction between the host polymers and the added NPs. In our previous work, we observed small peaks with weak intensity for the chitosan polymer incorporated with various amounts of alumina NPs [39,40]. 

### 3.2. Absorption Study

**Figure 3:**
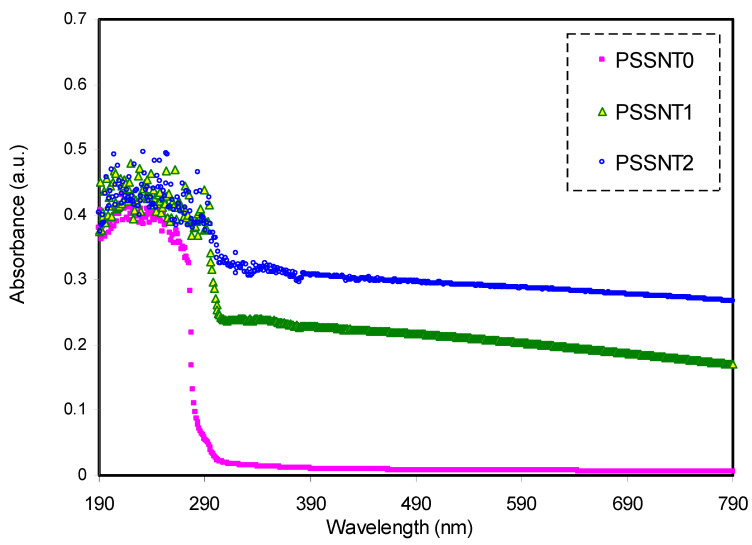
Absorption spectra of pure PS (PSSNT0), PSSNT1, and PSSNT2 nanocomposite films.

The absorption spectra of the pure PS and NCs were acquired to deal with the structure in terms of existing double bonds. It is noted that the inclusion of filler particles into functional polymer produced NCs with unique optical and dielectric properties, as confirmed in the previous work [41]. Figure 3 shows the pure PS and PS composites’ absorption spectra. It is apparent that in the absorption spectrum of the NC samples, there is a distribution of characteristic absorption over all the UV regions. It is noticed that the shoulder within the 200–400 nm range could be the reason for the association interaction between the neighboring phenyl groups in the PS matrix. The PS chains are flexible as there is an allowance of two free rotating phenyl groups around the carbon-carbon bond. The exposure of the samples to the UV increases the absorption over the 280–320 nm range, showing the absorbing photoproduct (i.e., the conjugated double bonds) in the PS [29]. The addition of nanofillers into the PS resulted in the shifting of absorption to higher wavelengths. This is because new states are introduced to the band gap (BG), and as a result the transition of electrons occurs from VB to CB. To gain more information on this phenomenon, the absorption edge can be studied. Basically, it is well known that if the incident photons’ energy is smaller than the difference in energy between the two levels of electrons, the energy of the photons will not be absorbed; thus, the material would be transparent to the photon. In contrast, the absorption of high energy photon occurs within 10 to 15 s, and the valence electrons jump between two energy levels of electrons [9]. The plateau feature of the absorption spectra in the visible region emphasizes the transparency of the PS composites for these photons, in which electrons are unable to transfer through the band structure [27].

### 3.3. Absorption Edge Study

The optical absorption spectrum is informative in terms of the energy band gap (BG) of amorphous and crystalline materials. It is best to use the absorption corresponding to the excitation of electrons from the VB to the CB to tackle the nature and quantification of the energy BG [42]. From an absorption study in which materials enable the absorption of light, the quantity is measured from the optical absorption coefficient (α) [43]. It is also essential to recognize the vibrational bands and the transitions of electrons in energy levels from the optical investigation. The absorption edge is defined as a region in which electrons jump from a state with low energy to a state with higher energy as a result of photon absorption [28].

Ultraviolet-visible (UV-Vis) spectroscopy is acknowledged as a proper technique for identifying the energy gap values within the materials. In the analysis of both the transmittance (*T*) and reflectance (*R*) spectra of the films, the *α* can be obtained [44]. The physical meaning of α is the fractional attenuation in intensity per unit distance, and the mathematical expression is seen below [45]:

(2) $$\alpha \;=\;\frac{-1}{I}\frac{dI}{dx}=\;\left(\frac{2.303}{d}\right)\;\times \;A$$

where *I* refers to the light intensity. The α is rearranged as follows,

(3) $$\alpha =\frac{1}{t}\ln\left(\frac{T}{1-R^{2}}\right)$$

where *t* refers to the samples thickness.

**Figure 4:**
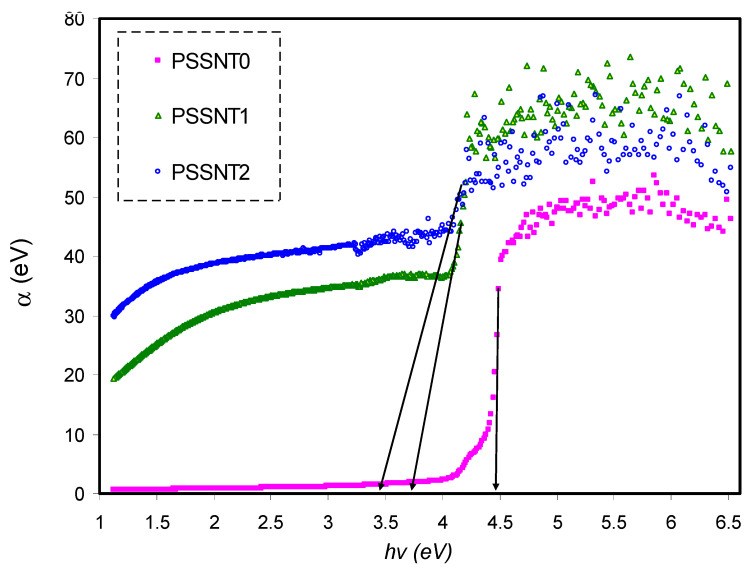
Absorption coefficient as opposed to photon energy for pure PS (PSSNT0), PSSNT1, and PSSNT2 nanocomposite films.

Figure 4 shows the relationship between *α* versus photon energy for the whole samples. Table 3 presents the values of the absorption edge. Interestingly, it is seen that the absorption edge value shifts from 4.4 eV for pure PS to 3.4 eV for the PS inserted with 8 wt % of SnTiO_3_. As previously stated, the characteristic absorption boundary region can be used to determine the minimum photon energy that is required to transfer the electrons from the VB to CB. Importantly, the BG can be determined by various methods and is supposed to be near the absorption edge value. The bond cleavage and reconstruction of formed conjugated bonds system result in the absorption edge shifting [28].

### 3.4. Refractive Index Analysis

At present, it is of great importance to examine the electrical and optical properties of polymers in large-scale uses for optical devices with considerable interference, polarization, reflection, and antireflection characteristics. It is interesting to modify the optical properties of polymers through the addition of dopants and taking into consideration their compatibility with the host matrices [13,15,46]. Among these properties, the refractive index (*n*) is essential to be dealt with as the measure of rate attenuation of the light speed in the medium. Refractive index (*n*) is a fundamental physical quantity of a material that shows the propagation of an electromagnetic wave within a medium, as shown below [14]:

(4) $$n=\;\frac{c}{v}$$

where *υ* and *c* refer to the velocity of light in a medium and the speed of light in space, respectively. When materials absorb light, the equation of the refractive index is modified as follows [14]:

(5) N=n+iK

where *N* refers to the complex refractive index; *n* refers to the real refractive index; and *K* refers to the extinction coefficient, which is related to the absorbed light [14].

To calculate the *n* of the films, the *R* and *K* can be used [15].

(6) $$n=\frac{1+R}{1-R}+\sqrt{\frac{4R}{{\left(1-R\right)}^{2}}-K^{2}}$$

**Figure 5:**
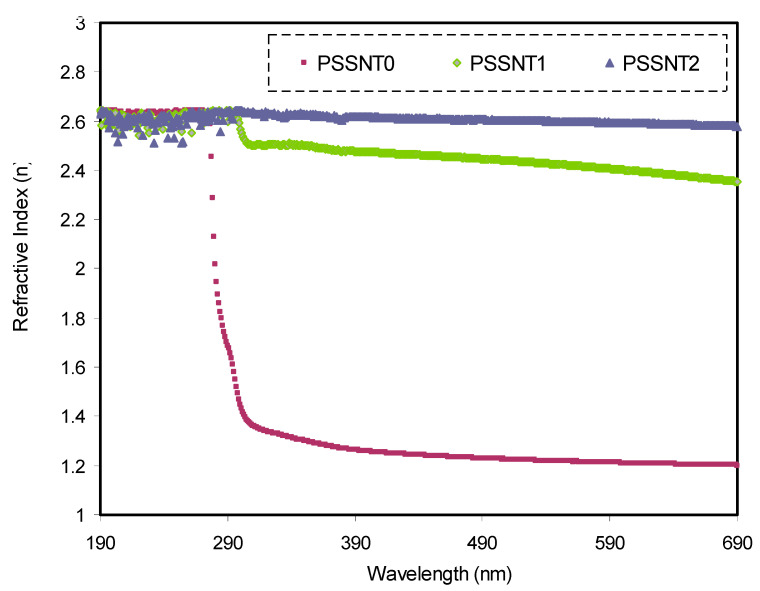
Refractive index spectra versus wavelength for pure PS (PSSNT0), PSSNT1, and PSSNT2 composite films.

It is clear that there is a direct relation among *K*, wavelength (*λ*), and *α*, whereas there is an inverse relation between *K* and *t* through K=αλ/4πt. Another mathematical expression exists in which the *R* is calculated using the values of *T* and absorbance *A* (R=1−(A+T)) + *T*). To compute the values of *T*, Beer’s law (T = 10^−A^) is used. The *n* spectra of pure PS and the doped samples are indicated in Figure 5. One can note that *n* is directly proportional to the SnTiO_3_ amount. It is also observed that the *n* value is bigger than pure PS as a consequence of photon deceleration from the interaction with the electrons of the host material.

To explain this phenomenon, i.e., the passage of light ray between a medium of air and a medium of a solid, we look at some of the consequences that will occur. The consequences include light reflection, absorption, and transmission through the medium. Accordingly, the incident light intensity to the second medium (*I_o_*) surface must be equivalent to the summation of the transmitted, absorbed, and reflected intensities that are denoted as *I_T_*, *I_R_*, and, *I_A_*, respectively, and it is mathematically expressed as follows:

(7) $$I_{o}=I_{R}+I_{A}+I_{T}$$

It is well known that the intensity of radiation is estimated in W/m^2^ and stands for the transmission of energy per unit time per unit area which is perpendicular to the propagation direction. Consequently, if (7) is divided by *I_o_*, the following formula is obtained:

(8) R+A+T=1

where *R, A*, and *T* refer to the reflectance equal to *I_R_/I_o_*, while the absorbance is equal to *I_A_/I_o,_* and the transmittance is equal to *I_T_/I_o_*, correspondingly. In other words, these are fractions of reflected, absorbed, and transmitted incident light. Based on these explanations, the entire light incident is subjected to reflection, absorption, or transmission, and their summation must be equivalent to 1 [8]. The *n* of a medium is the measure of the attenuation of the speed of light inside the medium. Its calculation is achieved using *R* and *K* of the films [14,28]. Therefore, the considerable *n* value of the composite systems means phase velocity deceleration. This is due to the SnTiO_3_ incorporation to the films, which results in a density increment, and as a consequence, *n* increases [28]. The obtained *n* values as illustrated in Figure 5 indicate that the insertion of the SnTiO_3_ filler into the PS polymer matrix can alter the refractive index of the NC films and as a result increase the *n* value from 1.23 to about 2.6.

The n reveals a polymer’s ability to reflect and bend light. The magnitude of n depends on the material chemical composition; for instance, 1.0 and 1.33 are documented for air and water, respectively [14]. Furthermore, the n of most titanium oxide pigments and polymers are 2.5 and 1.5, respectively. The *n* value of 1.3 to 1.7 for traditional polymers was reported in [47], while a higher value of *n* was documented for most inorganic materials [48,49]. The values of *n* achieved in this work are compared with those in the previous studies as shown in Table 4. Jin et al. [50] documented that the *n* of poly(methyl methacrylate) (PMMA) was enhanced from 1.49 to 1.839 upon the insertion of 20 wt % TiO_2_, which is smaller than that achieved in this study for the PS doped with SnTiO_3_ NPs. In our earlier study, we found an increase of *n* from 1.14 (pure PVA) to 2.25 for the PVA incorporated with 12 wt % of NaNO_3_ salt [51]. Therefore, the outcomes of this study show that a small quantity of filler instead of salts can improve *n* significantly. Due to the extensive uses of optical materials in the camera lens, glass lens, optical reflectors, optical waveguides, etc., high-refractive-index nanocomposites have been broadly considered. The advantages of organic materials are their cost efficiency, lightweightedness, transparency, ease of the process, and good mechanical properties, and the disadvantage is that they usually show a small refractive index [50]. The results of our work reveal that the ceramic nanofiller is unique to fabricate polymer NCs with a tuned refractive index.

### 3.5. Optical Dielectric Constant Study

**Figure 6:**
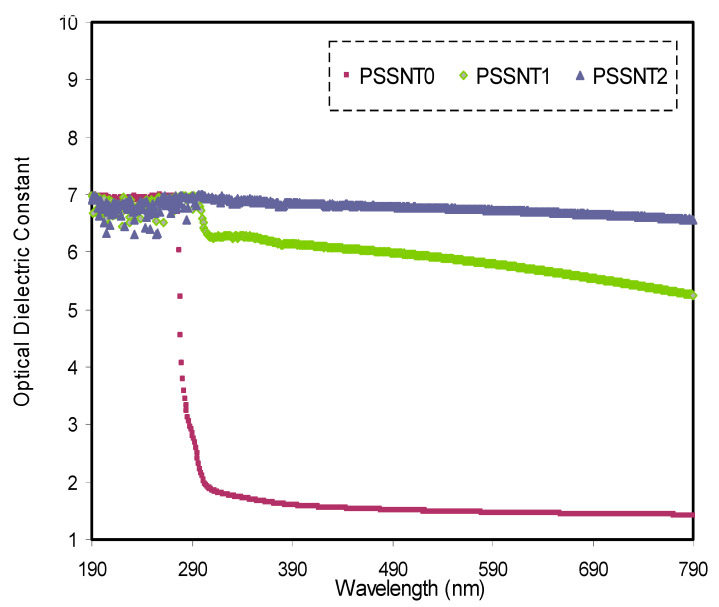
Dielectric constant spectra versus wavelength for pure PS (PSSNT0), PSSNT1, and PSSNT2 composite films.

It is proven that there is a relation between *n* and parameter of dielectric constant (ε′) to a large extent that is directly associated to the electron states’ localization inside the materials forbidden gap [27,34,54]:

(9) $$\varepsilon_{1}=n^{2}-k^{2}=\varepsilon_{\infty }-\frac{e^{2}}{4\pi C^{2}\varepsilon_{0}}\frac{N}{m^{*}}\lambda^{2}$$

where $\varepsilon_{\infty }$ and $\varepsilon_{0}$ refer to the dielectric constant at large wavelengths and the dielectric constant of the vacuum, respectively. The ratio between the localized density of electron states and the effective mass is denoted with N/m*. Figure 6 shows the relationship between ε′ and the wavelength at various SnTiO_3_ amounts. It is seen that the ε′ value and SnTiO_3_ concentration show a direct proportionality. It is crucial to note that increasing the ε′ value from 1.3 to 6.8 can be correlated to the additions of the density of states. These findings are based on this direct association between ɛ′ and the density of states within the composite polymer films’ forbidden gap [54]. It is common that there is a good correlation between the static dielectric constant ($\varepsilon_{0}$) and long wavelengths [55]. The Penn model states that there is a strong relation between ε′ and the optical band gap (*E_o_*) in this manner [56]:

(10) $$\varepsilon_{\left(o\right)}\approx 1+{\left(\hbar \omega_{p}/E_{o}\right)}^{2}$$

Moreover, according to this model, the *n* is associated with the ε′ (ε′ = *n*^2^). Therefore, the Penn model could be formulated in terms of *n* [57].

### 3.6. Band Gap Study

The BG energy is an energy range without electronic levels in a material. These levels lie between the conduction band (CB) and valence band (VB), which prevent any transition, i.e., it possesses forbidden energy. To create transitions between these two bands of energy, it required adequate energy, which is equivalent to the forbidden energy. As a result, electrons can transport freely as mobile carrier species between the VB and CB [58]. A deep understanding of the electron transition nature in charge-transfer complexes and semiconducting polymers has not been achieved yet. For the electron transfer occurrence from VB to CB, the energy of the incident photon must be higher than the forbidden energy, and the phonon needs to offer the necessary momentum [59]. In this study, two techniques were used to determine the BG values, which are Tauc’s model and optical dielectric loss parameter (ε″). The ε″ parameter was employed owing to the well-known formula on the basis of quantum mechanical accomplishments in which ε″ is related to valence and conduction bands.

Moreover, Tauc’s model is also a well-known method to measure BG values and identify the electron transition types with the aid of the ε″ technique. Earlier documents [2,60] designated that four plots can be drawn from Tauc’s model, and thus it is difficult to identify the correct transition as can be seen in a later section. In our previous studies [27,61], we confirmed that ε″ should be analyzed and compared with Tauc’s model to specify the type of electron transition.

#### 3.6.1. Optical Dielectric Loss Study

The concept of the interband absorption process originated in the electron transition between the bands in solid materials. Absorption edge appearance results from the optical transitions within the BG [62]. Previous studies showed that the fundamental absorption edge represents the energy BG originated from the dielectric loss (ε″) parameter [61,63,64]. This achievement is supported by quantum methods in the BG investigation. From both ε′ and ε″ parameters, we are allowed to calculate the fundamental optical functions [65]. To know the electronic structure of materials, it is of great importance to determine the optical functions. Here, it is worth correlating the frequency-dependence dielectric function to the electronic band structure.

Based on these explanations, at all photon energy ranges, the optical properties of homogeneous mediums can be quantified [66]. From the principle of quantum mechanics, the ε″ parameter is strongly associated with the transitions between valence and conduction bands [61,63,64,65,66]. The ε′ is interrelated to the material’s electronic polarizability, and the ε″ is correlated to an electronic absorption of the materials [67]. Generally, in terms of the analysis of the complex dielectric function (ε*), the optical properties can be interpreted and mathematically shown below:

(11) $$\varepsilon_{\left(\omega \right)}^{*}\;=\;\varepsilon^{\prime }\;+\;{i\varepsilon }^{\prime \prime }$$

**Figure 7:**
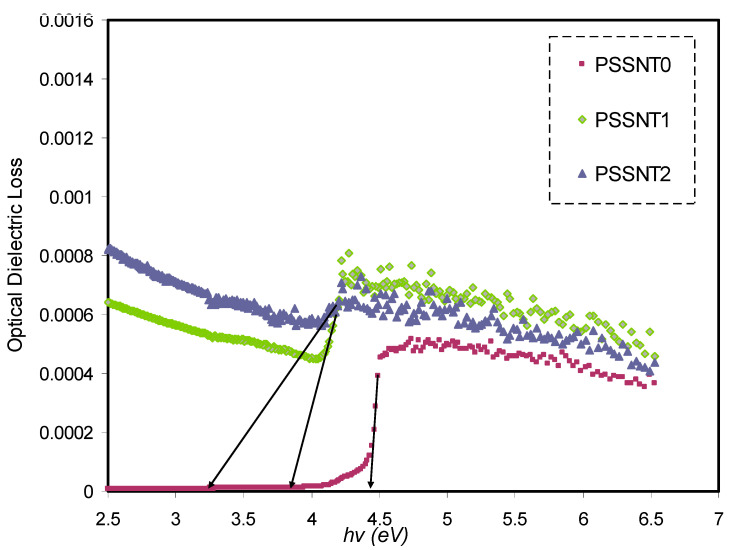
The optical dielectric loss (ε″) spectra versus photon energy for pure PS (PSSNT0), PSSNT1, and PSSNT2 composite films.

**Figure 8:**
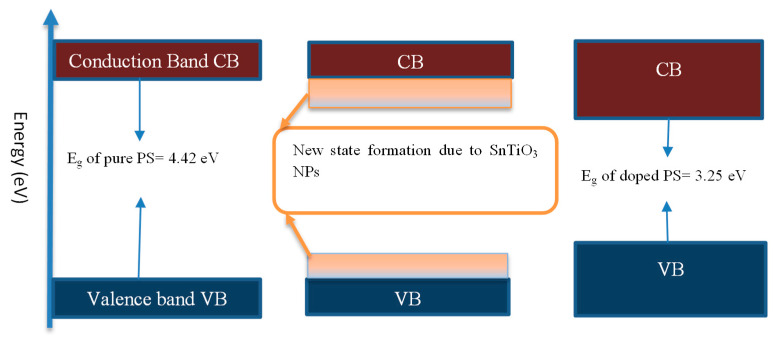
Nanofiller effect on the band gap reduction.

As a fact, the indirect and direct BG transitions are involved in ε^*^_(ω)_, although with indirect BG transitions there is a minor involvement to ε^*^_(ω)_ compared to the direct transition, and both are mediated by phonons [68]. It is recognized that estimating the exact BG energy is impossible using Tauc’s equations because of the disposable constant numbers in the formulations [27,46,63,64,69]. A new hypothesized method is proposed to examine the exact BG energy. This is proven based on the data analysis [27,63,64,69]. This initial study provides a way to recognize the parameter of ε″ as a crucial component to determine the optical band gap accurately. This postulation is based on the recent developments in quantum mechanics and on the illustrations established from the study of the BG. Determination of the ɛ” by the elements of momentum matrix from the filled electronic states to the unfilled electronic states is obtained from the following equation:

(12) $$\varepsilon^{\prime \prime }=\frac{2\pi e^{2}}{\Omega \varepsilon_{o}}\underset{v,c,k}{\sum }{\left|\psi_{k}^{c}\left|\vec{u}.\vec{r}\right|\psi_{k}^{v}\right|}^{2}\delta \left(E_{k}^{c}-E_{k}^{v}-\hbar \omega \right)$$

where ω refers to the light frequency; $\vec{u}$ vector refers to the polarization of the incident electric field; $\left(E_{k}^{c}\right)$ and $\left(E_{k}^{v}\right)$ refer to the wave function of CB and the wave function of VB at *k*, respectively; and *e* has its usual meaning [61,63]. It is well documented that the absorption edge in ε″ spectra is used to provide the BG energy [70]. It is straightforward to use the rapid rise near the absorption edge directly in the ε″ spectra to determine the BG value [16,71]. The fundamental absorption edge that appears in the optical spectra originates in the top of the VB to the bottom of the CB. Thus, the BG values obtained in this analysis are critical points [72]. According to these explanations, the BG is defined by the absorption edge [70]. In other words, the obtained BG values from the analysis of significant points in the ε″ spectra are intimately correlated [72]. The optical ε″ plot versus the energy of the photons for all the films is shown in Figure 7. The optical band gaps are obtained from the interception between the linear part of ε″ and the axis of the photon energy. The SnTiO_3_ NPs role on the BG reduction is indicated in Figure 8. It is believed that filler insertion may introduce additional levels inside the BG, and therefore, these new levels will ease the electrons’ transport from the VB to the CB.

#### 3.6.2. Tauc’s Model

**Figure 9:**
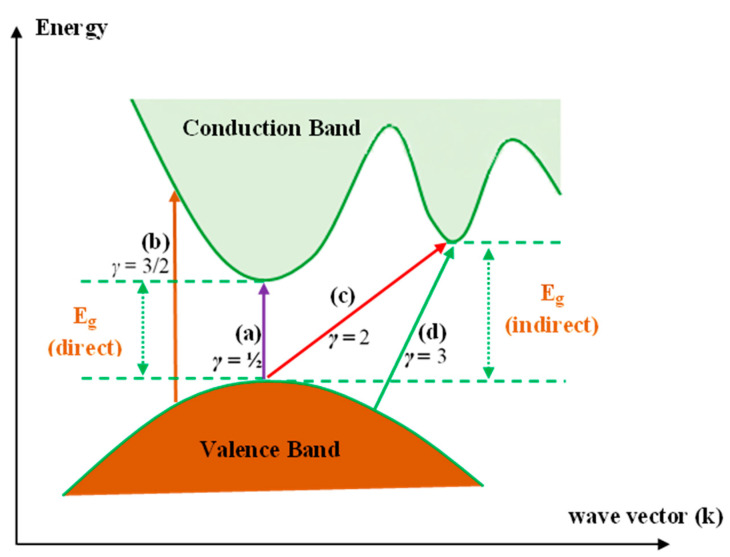
Transition types: (a)—direct allowed, (b)—direct forbidden, (c)—indirect allowed, and (d)—indirect forbidden [75].

The optical properties are analyzed from the response of the materials due to the exposure to electromagnetic radiation, particularly visible light. From a quantum mechanical viewpoint, the electromagnetic radiation is considered as a form of energy named photons instead of waves [8]. The photon energy is quantized through Planck’s law:

(13) E = hυ = hc/λ

where *h* refers to the Planck constant which is equal to 6.63 × 10^−34^ J/s, *c* refers to the light speed in space, and *λ* refers to the photon wavelength. As a fact, when a light wave propagates through materials, it experiences attenuation with distance. Photons have the capability for exciting electrons from the occupied levels in the VB to the unoccupied levels in the CB. This procedure is purely quantum mechanical in nature and is known as interband transitions [27,63,64,69]. The analysis of the α data in response to the wavelength in the evaluation of the optical band gap is found to be dependent on Tauc’s relation [61,63],

(14) $$\left(\alpha hv\right){\;}^{1/\gamma }=\;B\left(hv\;-\;E_{g}\right)$$

where *hυ* refers to the photon energy (eV), *B* stands for the band form parameter, *E_g_* stands for the optical energy BG, and *γ* = ½ and 2 refer to the direct allowed and indirect allowed transitions, respectively. It is also worth mentioning that *γ* takes the values of 3 and 3/2 for the indirect forbidden transition and direct forbidden transition, respectively [2,8,60]. Figure 9 indicates various kinds of electronic transition that can happen between the VB and CB based on Tauc’s model [73,74].

Based on the solid-state physics view, there is a range of energy in a solid material that signifies the energy gap where the energy states are not permitted. This implies that there is a lack of absorption of free charge carriers, and the interband transitions are restricted to cases of photons with higher energies [76].

**Figure 10:**
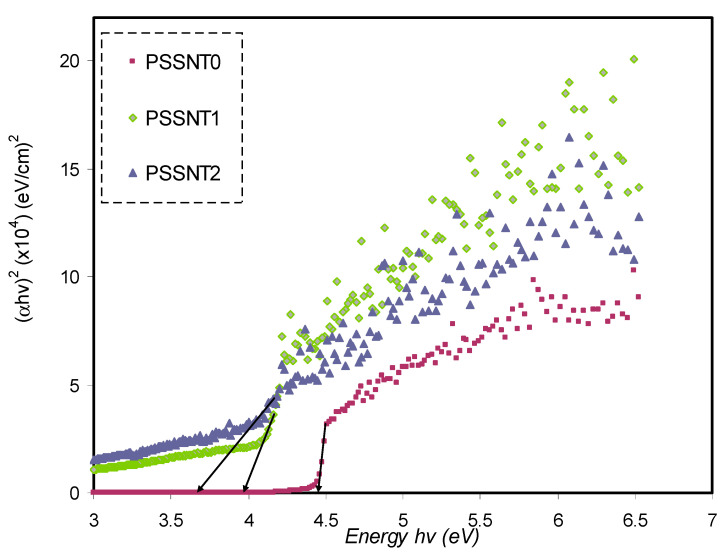
Plot of (*αhυ*)^2^ versus photon energy for pure PS (PSSNT0), PSSNT1, and PSSNT2 NC films, where *α* represents the absorption coefficient.

**Figure 11:**
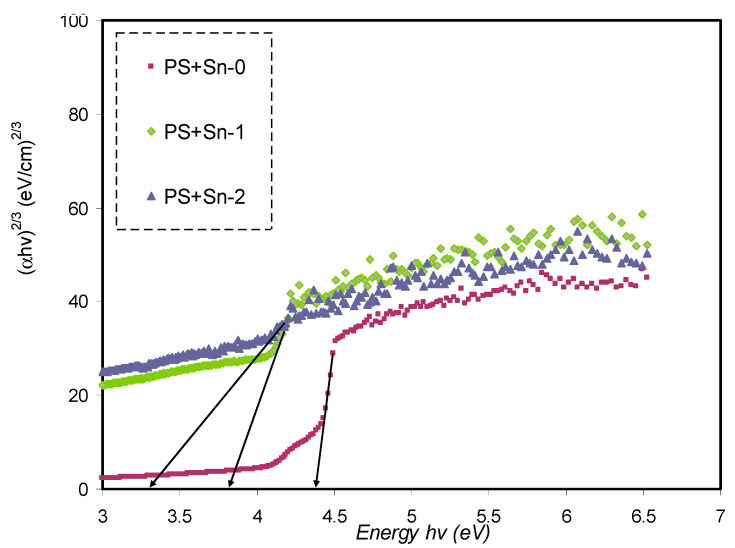
Plot of (*αhυ*)^2/3^ versus photon energy for pure PS (PSSNT0), PSSNT1, and PSSNT2 NC films.

**Figure 12:**
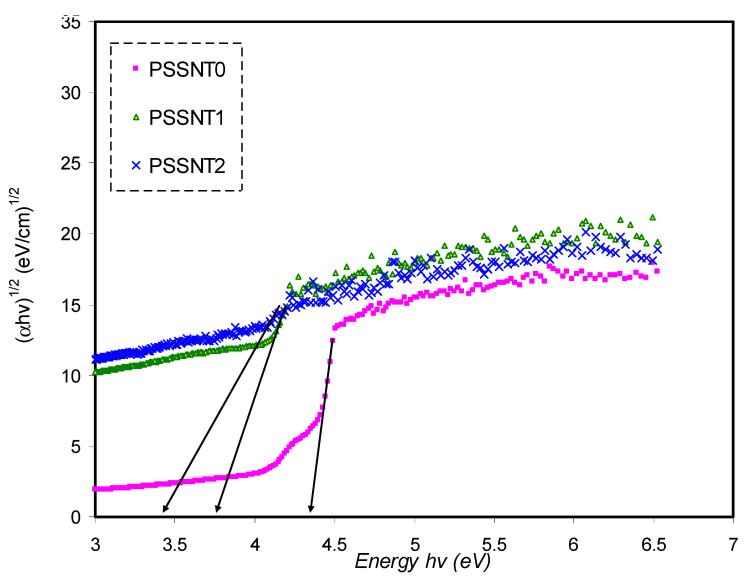
Plot of (*αhυ*)^1/2^ versus photon energy for pure PS (PSSNT0), PSSNT1, and PSSNT2 NC films.

**Figure 13:**
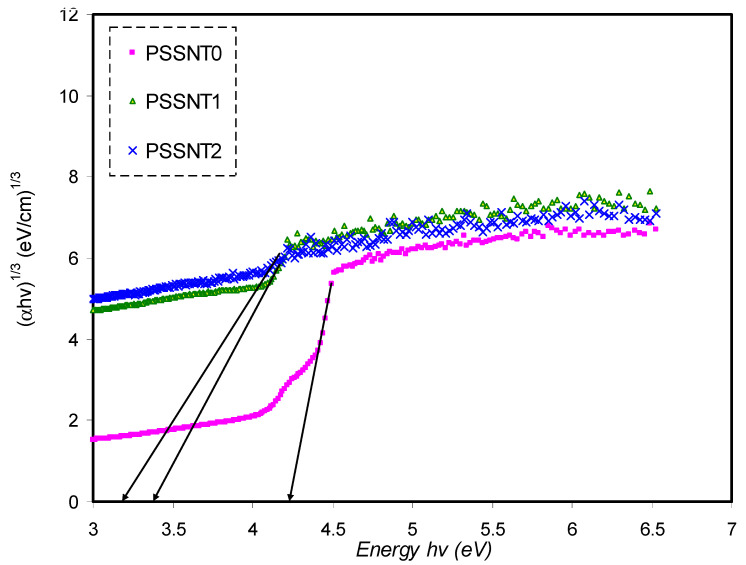
Plot of (*αhυ*)^1/3^ versus photon energy for pure PS (PSSNT0), PSSNT1, and PSSNT2 NC films.

The plots of (*αhυ*)^1/γ^ versus *hυ* for four values of *γ* are presented respectively in Figure 10, Figure 11, Figure 12 and Figure 13. In determining the optical BG of the samples, the curves’ linear parts close to zero absorption value are extrapolated and tabulated in Table 5. 

We noticed that the evaluated BG values from *γ* values decrease with the incorporation of SnTiO_3_. As a result, the use of Tauc’s relation in all plots for all *γ* values is not helpful in the determination of the types of electronic transition. Therefore, to determine the types of electronic transition and BG accurately, the parameter of ε″ must be considered. From the view of the principle of quantum mechanics, the photon and electron interaction in the system is interpreted based on the ground electronic state time-dependent perturbations. As a principle, the photon emission and absorption cause transitions between the filled levels and unoccupied levels [77].

The electronic transition and charge transfer are not fully understood in semiconducting/conducting polymers. Nevertheless, by producing necessary momentum and energy from the photon and phonon incident, the transitions occur [59]. Optical band gap determination and electronic transition types can be performed respectively from the optical ɛ″ parameter and Tauc’s method, as emphasized in previous studies [2,8,14,61,63]. This is because it is hard to relate the band structure to the optical dielectric function in the materials. To be accurate in establishing this association, this would need appropriate attention to complex dielectric function (ɛ*) quantum physics. It is well known that the electronic response of material electron density to incident radiation of electromagnetic is discussed in terms of the ɛ* [78]. To pinpoint, from Tauc’s equation, it is difficult to predict whether the structure of the band is either a direct type or an indirect one, as explained earlier [79]. The actual transition from the filled levels to the unfilled levels could be explained from the ɛ″ parameter [80].

**Figure 14:**
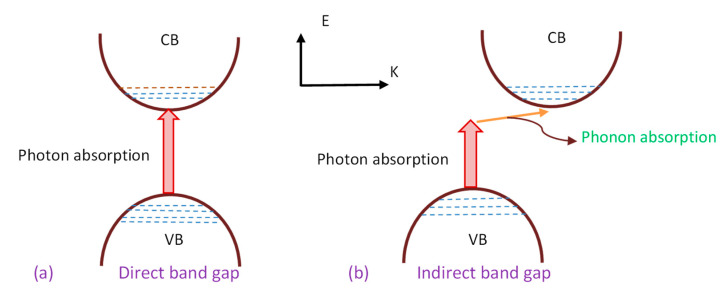
Electronic transition with (**a**) direct allowed transition and (**b**) direct forbidden transition.

It is seen in Table 5 that the measured energy gap (*E_g_*) values for PS composite films for γ = 3/2 (direct forbidden) are relatively close to the *E_g_* values attained from the ε″ plot (Figure 7), indicating that the crystalline nature was perturbed in the PS composite films. Thus, the type of electron transitions in the PS composite films is a direct forbidden one (γ = 3/2). The *E_g_* values obtained from Figure 10, Figure 11, Figure 12 and Figure 13 compared to the obtained *E_g_* values using the ε″ plot (Figure 7) indicate that electron transitions in pure PS are direct allowed (γ = ½). Based on ε″ and Tauc’s method results, the type of electron transition in pure PS is a direct transition, as shown schematically in Figure 14a. In contrast, the direct forbidden transition is dominant in the incorporated samples, and their transitions are shown schematically in Figure 14b.

## 4. Conclusions

In conclusion, the optical dielectric parameter is a crucial parameter for BG investigations of materials. The SnTiO_3_ NPs impact the PS structure in the films, and it was found that the hump of PS decreased when increasing the NPs. The prominent sharp and high-intensity peaks resulted from the high incorporation of SnTiO_3_ NPs. The absorption spectra were altered with increasing SnTiO_3_ nanoparticles, where the absorption edge shifted to smaller photon energy sides up on the NPs inclusion to the PS host polymer. In addition, the *n* and ε′ relationship was established. The *n* and ε′ significantly increased upon the inclusion of SnTiO_3_ nanoparticles to the PS polymer. Tauc’s method was employed to investigate the electron transitions’ nature in pure PS and composite materials. The ε″ was precisely investigated to compute the energy band gap. The nature of electron transitions in pure PS was found to be direct allowed, while it was found to be direct forbidden for the PS nanocomposite films. Because of their optical energy BG and flexibility, these films have enormous possibilities for extensive application in optoelectronic devices.

## Figures and Tables

**Table 1:** Nanoparticle size *D* determination using Debye-Scherrer’s equation.

2θ Degree	cos θ	*D* (nm)	*D*_Average_ (nm)
30.85°	0.963	20.85	25.179
32.15°	0.96	33.19	
44.05°	0.927	24.91	
45.05°	0.923	21.76	

**Table 2:** Full width at half maximum (FWHM) for pure polystyrene (PS) and nanocomposite films.

Sample Code	FWHM
PSSNT0	0.963
PSSNT1	0.96
PSSNT2	0.923

**Table 3:** Pure PS and PS nanocomposites absorption edge values.

Sample Code	Absorption Edge (eV)
PSSNT0	4.4
PSSNT1	3.7
PSSNT2	3.4

**Table 4:** Measured refractive index for different polymer composites.

Composition	Refractive Index	Ref.
PMMA: TiO_2_	1.839	[50]
PGMA: TiO_2_	1.8	[52]
PMMA: ZnO	≈1.65	[53]
PVA: Al powder	2.14	[13]
PVA-Cu(II)-complex	1.6	[14]
PS: SnTiO_3_	2.6	This work

**Table 5:** Band gap *E_g_* from Tauc’s method (*αhυ*)^1/γ^ versus photon energy *hυ* and the optical dielectric loss ε″ plot, where *α* represents the absorption coefficient.

Sample Code	*E_g_* for *γ* = 1/2	*E_g_* for *γ* = 2	*E_g_* for *γ* = 3	*E_g_* for *γ* = 3/2	*E_g_* from *ɛ_i_* plot
PSSNT0	4.44	4.39	4.24	4.35	4.42
PSSNT1	3.94	3.75	3.38	3.76	3.78
PSSNT2	3.64	3.4	3.2	3.27	3.25
